# Human Cytomegalovirus US28 Facilitates Cell-to-Cell Viral Dissemination 

**DOI:** 10.3390/v6031202

**Published:** 2014-03-12

**Authors:** Vanessa M. Noriega, Thomas J. Gardner, Veronika Redmann, Gerold Bongers, Sergio A. Lira, Domenico Tortorella

**Affiliations:** 1 Department of Microbiology, Icahn School of Medicine at Mount Sinai, One Gustave L. Levy Place, New York, NY 10029, USA; E-Mails: Vanessa.Noriega@mssm.edu (V.M.N.); Thomas.Gardner@mssm.edu (T.J.G.); 2 Department of Pathology and Immunology, Washington University School of Medicine, 660 South Euclid Avenue, St. Louis, MO 63110, USA; E-Mail: vredmann@pathology.wustl.edu; 3 Immunology Institute, Icahn School of Medicine at Mount Sinai, One Gustave L. Levy Place, New York, NY 10029, USA; E-Mails: Gerold.Bongers@mssm.edu (G.B.); Sergio.Lira@mssm.edu (S.A.L.)

**Keywords:** human cytomegalovirus, BAC recombineering, viral GPCR US28, virus dissemination, virus growth, membrane protein biology

## Abstract

Human cytomegalovirus (HCMV) encodes a number of viral proteins with homology to cellular G protein-coupled receptors (GPCRs). These viral GPCRs, including US27, US28, UL33, and UL78, have been ascribed numerous functions during infection, including activating diverse cellular pathways, binding to immunomodulatory chemokines, and impacting virus dissemination. To investigate the role of US28 during virus infection, two variants of the clinical isolate TB40/E were generated: TB40/E-US28^YFP^ expressing a C-terminal yellow fluorescent protein tag, and TB40/E-FLAG^YFP^ in which a FLAG-YFP cassette replaces the US28 coding region. The TB40/E-US28^YFP^ protein localized as large perinuclear fluorescent structures at late times post-infection in fibroblasts, endothelial, and epithelial cells. Interestingly, US28^YFP^ is a non-glycosylated membrane protein throughout the course of infection. US28 appears to impact cell-to-cell spread of virus, as the ΔUS28 virus (TB40/E-FLAG^YFP^) generated a log-greater yield of extracellular progeny whose spread could be significantly neutralized in fibroblasts. Most strikingly, in epithelial cells, where dissemination of virus occurs exclusively by the cell-to-cell route, TB40/E-FLAG^YFP^ (ΔUS28) displayed a significant growth defect. The data demonstrates that HCMV US28 may contribute at a late stage of the viral life cycle to cell-to-cell dissemination of virus.

## 1. Introduction

Human cytomegalovirus (HCMV) is a widespread pathogen that infects a vast majority of the world’s population [1]. HCMV is the prototypic β-herpesvirus, characterized by its extended replication cycle, restricted host range, and cytopathic effect of pronounced cell swelling [2]. Infection of the healthy, immunocompetent host is typically asymptomatic, with pressure from the immune system leading to establishment of lifelong latent infection within cells of the myeloid lineage [3]. Infection of the immunologically immature or reactivation of latent infection during times of immunosuppression can result in significant disease [4]. In fact, HCMV infection during solid organ or hematopoietic stem cell transplant can have severe implications for the host and can ultimately prove fatal [5]. 

The exceptionally large HCMV genome encodes for over 200 genes [6], including four putative homologs of cellular G protein-coupled receptors (GPCRs): the HCMV-specific US27 and US28, and the β-herpesviruses-conserved UL33 and UL78 [7]. GPCRs, also known as seven-transmembrane domain proteins, are integral membrane receptors that sense extracellular ligands to trigger signal transduction networks and coordinate cellular responses [8]. Once activated, these receptors undergo a conformational change, causing activation of an associated heterotrimeric G protein and leading to production of intracellular secondary messenger molecules to induce downstream signaling pathways. HCMV infection is known to modulate a number of host cellular responses, including intracellular calcium levels, cyclic AMP (cAMP) production, inositol phosphate hydrolysis, and activation of phosphatidylinositol-3-kinase (PI3K) [9]. As constituents of the virion [10,11,12], several of the HCMV‑encoded GPCRs regulate a number of these pathways immediately following infection. Both US28 and UL33 signal constitutively and can alter inositol phosphate production and activation of NF‑κB and cAMP response elements (CRE) [13,14]. Furthermore, US28 can bind CC chemokines to induce increases in intracellular calcium levels and migration of infected cells [15,16]. Although it shows no constitutive activity [17], US27 was recently found to enhance signaling mediated by endogenous CXCR4, resulting in enhanced calcium mobilization and chemotaxis [18]. To date no activating ligands or signaling properties have been attributed to UL78.

Another intriguing characteristic accorded to HCMV-encoded GPCRs is their contribution to dissemination of virus *in vitro*. UL78 appears to impact virus growth in both endothelial and epithelial cells [12]. Additionally, UL78 supports infection by coordinating the timely delivery of viral DNA into the nuclei of infected cells [12]. US27 is required for efficient spread by the extracellular route and influences virus growth in fibroblasts and endothelial cells [19]. Expression of the murine cytomegalovirus (MCMV) ortholog M33 protein was shown to be necessary for virus dissemination *in vivo* but not in tissue culture [20]. An MCMV mutant lacking the GPCR M78 exhibited a growth defect in culture and reduced pathogenicity in mice [21]. The implication of HCMV-encoded GPCRs as virulence factors to enhance infection is quite intriguing, as their presence within infected cell membranes [22,23] could allow cell-cell communication and modulation of signaling networks within neighboring cells to facilitate propagation.

To determine the role of US28 in HCMV dissemination, mutational analysis of the TB40/E clinical isolate was performed. A YFP derivative of US28 (TB40/E-US28^YFP^) localized as large perinuclear structures at late times of infection in fibroblasts, endothelial, and epithelial cells. At these late times, US28^YFP^ was integrated into cellular membranes, further validating its presence at the interface of infected cells. A ΔUS28 mutant (TB40/E-FLAG^YFP^) produced increased levels of extracellular virus as assayed by both multi-step and single-step growth kinetics. Extracellular virus produced by the ΔUS28 mutant could be neutralized by the addition of HCMV glycoprotein-specific antibodies and spread of TB40/E-FLAG^YFP^ by the cell-to-cell route was abrogated in fibroblasts and epithelial cells. These findings implicate the viral GPCR US28 as a factor contributing to cellular dissemination of HCMV. 

## 2. Results

### 2.1. Generation of HCMV TB40/E US28 Variants

**Figure 1:**
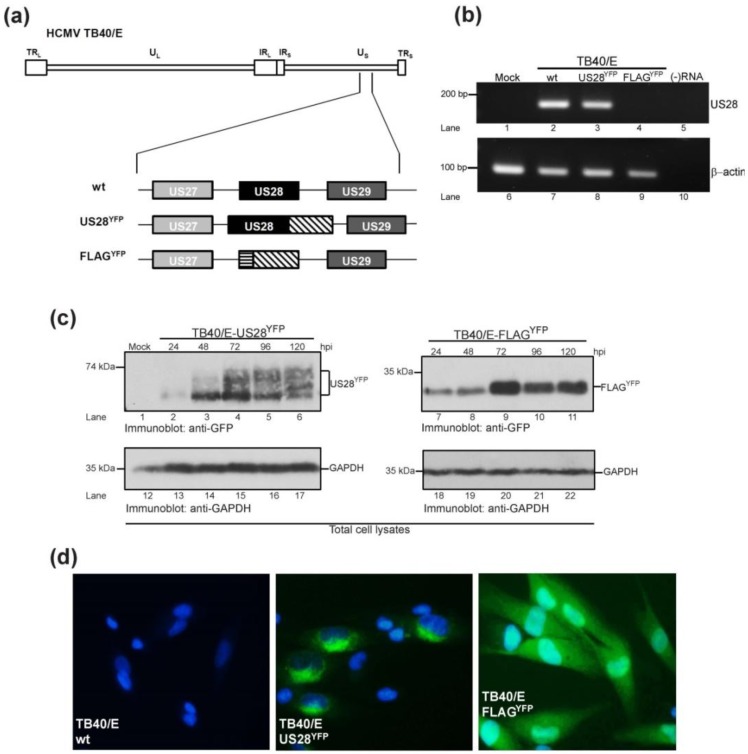
Generation of TB40/E-US28 variants. (**a**) Using a bacterial artificial chromosome (BAC) recombineering approach Human cytomegalovirus (HCMV) TB40/E variants were generated that express either chimeric US28 containing a carboxy-terminal YFP tag (US28^YFP^) or a US28 deletion mutant where the US28 ORF has been replaced with an engineered FLAG-YFP cassette (FLAG^YFP^). YFP sequences are denoted by the diagonally hatched box; FLAG sequences are denoted by the horizontally striped box. TR, terminal repeat; U, unique sequences; IR, inverted repeat; L, long; S, short. (**b**) Fibroblasts mock-infected or infected (MOI = 5) with TB40/E wt or TB40/E-US28 variants were harvested 48 hours post-infection and subjected to RT-PCR with primers specific to US28 (lanes 1–5) or β-actin (lanes 6–10). A sample lacking RNA ((−)RNA) was included as a negative control. HCMV US28, β-actin, and relative DNA standards are indicated. (**c**) Fibroblasts mock-infected or infected (MOI = 5) with TB40/E-US28^YFP^ or TB40/E-FLAG^YFP^ were harvested at the indicated times and subjected to SDS-PAGE and immunoblot analysis. US28^YFP^, FLAG^YFP^, GAPDH, and molecular weight standards are indicated. (**d**) Fibroblasts infected (MOI = 5) with TB40/E wt, TB40/E-US28^YFP^ or TB40/E-FLAG^YFP^ were harvested 48 hours post-infection and visualized using the EVOS Cell Imaging Systems at 60× magnification.

To extend on studies of viral GPCRs as virulence factors, derivatives of the HCMV clinical isolate TB40/E were generated (Figure 1a). The wild type TB40/E bacterial artificial chromosome (BAC) (herein termed TB40/E wt) was altered to express a chimeric protein in which the carboxy terminus of the US28 coding region was amended with a yellow fluorescent protein tag (TB40/E-US28^YFP^) (Figure 1a). A second variant was generated in which the US28 coding region was replaced with a DNA cassette encoding a FLAG-tagged YFP chimera (TB40/E-FLAG^YFP^) (Figure 1a). To confirm abrogation of US28 message in the ΔUS28 (FLAG^YFP^) virus, MRC5 lung fibroblasts were mock‑infected or infected with TB40/E wt, TB40/E-US28^YFP^ or TB40/E-FLAG^YFP^ and RNA harvested at 48 hours post-infection, a time when US28 should be abundantly transcribed [24]. RT-PCR analysis with primers specific to a region within US28 demonstrated that US28 messenger RNA continued to be generated during infection with TB40/E wt and TB40/E-US28^YFP^, but not with the ΔUS28 virus (Figure 1b, lanes 1–4). To further confirm expression of our TB40/E YFP chimeras, fibroblasts were either mock-infected or infected with TB40/E-US28^YFP^ or TB40/E-FLAG^YFP^, harvested at various times post-infection, and analyzed by immunoblot for expression of YFP (Figure 1c). Kinetic analysis confirmed US28^YFP^ expression throughout the time course, with maximal expression at 72 hours post‑infection (Figure 1c, lanes 1–6). US28^YFP^ migrated as a broad polypeptide species of approximately 65 kD (Figure 1c, lanes 1–6). FLAG^YFP^ followed a similar time course of expression, peaking at 72 hours post-infection (Figure 1c, lanes 7–11). When visualized by fluorescence microscopy, the majority of US28^YFP^ localized intracellularly to vesicular structures concentrated around the nucleus (Figure 1d, center), confirming earlier data for US28 localization in transiently transfected cells [22]. A small portion of US28^YFP^ appeared to localize to the cell surface, as US28 undergoes constitutive endocytosis and recycling [22]. TB40/E-FLAG^YFP^-infected cells expressed fluorescence throughout the cell (Figure 1d, right) while the TB40/E wt parental virus did not express YFP (Figure 1d, left). Taken together, the data demonstrates that TB40/E variants of the US28 coding region had been generated to ascertain its role in HCMV virulence.

### 2.2. HCMV US28^YFP^ Localizes as Large Vesicular Structures at Late Times of Infection

**Figure 2:**
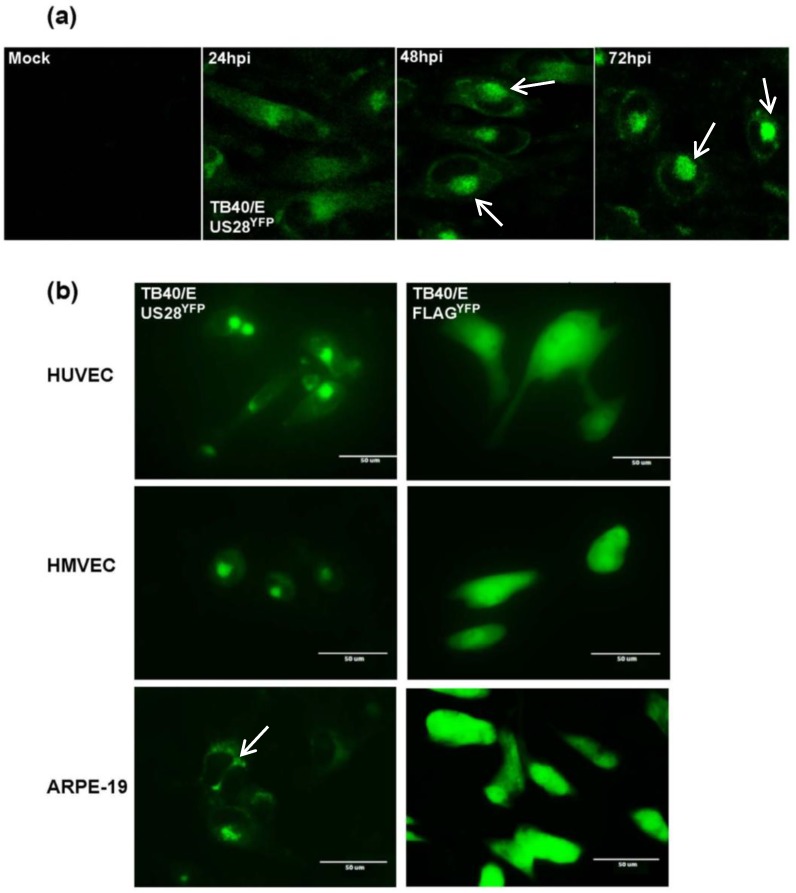
Formation of US28-expressing structures late during HCMV infection. (**a**) Confocal microscopy was performed on fibroblasts either mock-infected or infected with TB40/E-US28^YFP^ (MOI = 5). At various times post-infection cells were fixed and analyzed using an ImageXpress Ultra plate-scanning confocal microscope. (**b**) Endothelial and epithelial cells were infected with either TB40/E-US28^YFP^ (left) or -FLAG^YFP^ (right) (MOI = 25) and visualized at 4 days post-infection using the EVOS Cell Imaging Systems at 60× magnification.

To visualize a time course of US28^YFP^ expression, confocal microscopy was performed on fibroblasts infected with TB40/E-US28^YFP^ (Figure 2a). At early times post-infection, US28^YFP^ localized diffusely throughout the cell (Figure 2a, left). As infection progressed, US28^YFP^ coalesced into intense fluorescent perinuclear structures focused on one side of the nucleus (Figure 2a, 48, 72 hpi, arrows). By 72 hours post-infection, these large structures seemed to encroach on the nuclear space (Figure 2a, arrows). These organelles most likely represent viral assembly zones, as US28 has been proposed to be incorporated into assembling virions.

To determine if US28^YFP^ localization into large perinuclear structures late during infection was cell‑type specific, infections of human umbilical vein endothelial cells (HUVECs), human microvascular endothelial cells (HMVECs), and ARPE-19 epithelial cells were performed (Figure 2b). Infection with TB40/E-US28^YFP^ caused the formation of intense fluorescent granular structures in all cell types assayed (Figure 2b, left column). In comparison infection with TB40/E-FLAG^YFP^ resulted in diffuse fluorescence throughout the cell for each cell type (Figure 2b, right column). US28^YFP^ expression in endothelial cells was similar to cell surface staining on smooth muscles cells expressing US28, in which the viral GPCR accumulated toward the leading edge of migrating cells [16]. Strikingly, in epithelial cells, US28^YFP^ also appeared to converge at the junction of neighboring infected cells (Figure 2b, bottom left, arrow). Taken together, the results demonstrate that US28 localizes to large perinuclear structures that may represent areas of infectious virus production. 

### 2.3. Characterization of US28^YFP^ in HCMV-Infected Cells

#### 2.3.1. HCMV US28^YFP^ Is Integrated into Dense Vesicular Bodies

**Figure 3:**
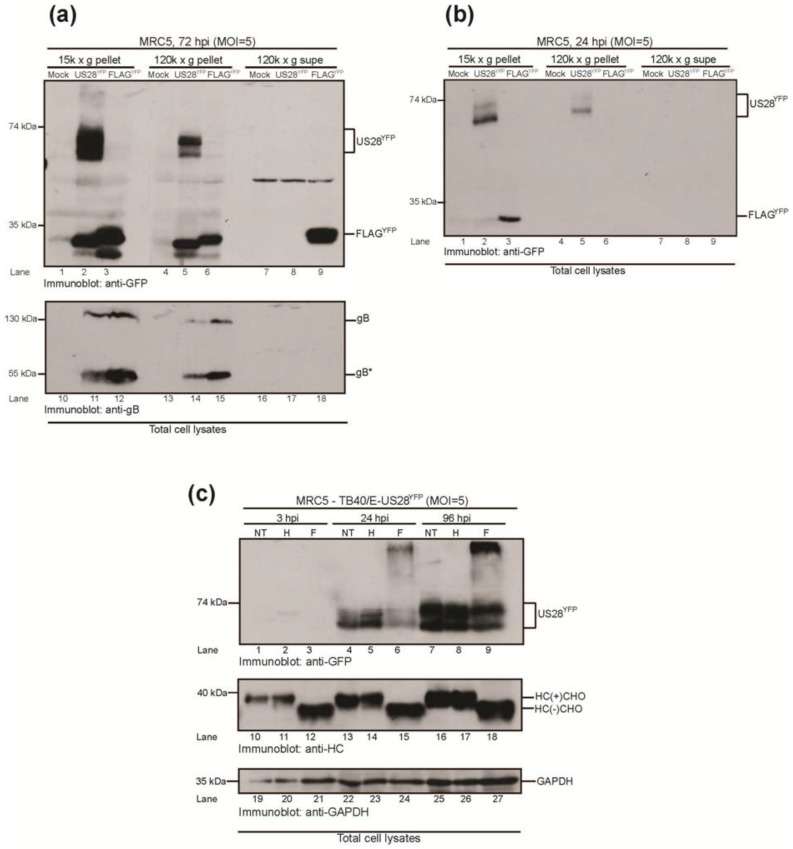
HCMV US28 is a non-glycosylated membrane protein incorporated into infected cells. Fibroblasts mock-infected or infected (MOI = 5) with either TB40/E-US28^YFP^ or TB40/E-FLAG^YFP^ were subjected to subcellular fractionation at 72 (**a**) or 24 (**b**) hours post-infection. Cell pellets from the 15,000 and 120,000 × *g* centrifugations (15 k × g pellet, 120 k × g pellet) and the 120,000 × *g* supernatant (120 k × g supe) were resolved by SDS-PAGE and subjected to immunoblot analysis. US28^YFP^, FLAG^YFP^, gB, and molecular weight standards are indicated. gB* indicates the mature form of glycoprotein B. (**c**) Fibroblasts infected with TB40/E-US28^YFP^ (MOI = 5) were harvested at the indicated time points and left non-treated (NT) or treated with EndoH (H) or PNGaseF (F). US28^YFP^, glycosylated MHC class I heavy chains (HC(+)CHO), deglycosylated MHC class I heavy chains (HC(−)CHO), GAPDH, and molecular weight standards are indicated.

The viral GPCRs US27 and UL33 localize to virus-wrapping membranes on HCMV-infected cells [23]. To determine if US28, at late time points post-infection, also traffics to dense vesicles consisting of large complexes, subcellular fractionation was performed on TB40/E-US28^YFP^- and TB40/E-FLAG^YFP^-infected fibroblasts (Figure 3a). At 72 hours post-infection, cells were lysed using a ball-bearing homogenizer and subjected to two centrifugation steps: nuclei and heavy/dense organelles were spun down at 15,000 × g, followed by separation of cellular membrane and cytoplasm by high-speed centrifugation at 120,000 × g. A substantial amount of US28^YFP^ and FLAG^YFP^ localized to the 15,000 × g fraction containing heavy organelles (Figure 3a, lanes 1–3). As US28^YFP^ and FLAG^YFP^ polypeptides are being abundantly synthesized at this late time point of infection (Figure 1c), their localization to this fraction may represent ER membranes contiguous with the nucleus, large protein complexes, and large dense membrane vesicles. Considering the levels of US28^YFP^ in the 15,000 × g fraction, only a small amount of two distinct US28^YFP^ species were localized to cellular membranes after high-speed centrifugation (Figure 3a, lane 5), suggesting that US28^YFP^ traffics with large protein complexes in dense membrane fractions. Interestingly, FLAG^YFP^ was also found in this membranous fraction (Figure 3a, lane 6). This may simply represent contamination from the cytoplasmic fraction, as the majority of FLAG^YFP^, and not US28^YFP^, localizes to the cytoplasm (Figure 3a, lane 8–9). As a control, immunoblot analysis of viral glycoproteins was also performed (Figure 3a, lanes 10–18). The glycoprotein gB also localized to the dense cellular membrane fraction (Figure 3a, lanes 10–15) and not the cytoplasmic fraction (Figure 3a, lanes 16–18). A similar result was found for the viral glycoprotein gH (data not shown). Remarkably, US28^YFP^ trafficked within dense cellular membranes as early as 24 hours post-infection (Figure 3b, lane 2) probably due to active translation on large ER membranes. Alternatively, the localization of US28^YFP^ to the 15,000 × g fraction may be due to integration of US28 from the HCMV virion into the plasma membrane following infection, but additional experiments are needed to confirm this. Taken together, the data demonstrates that US28^YFP^ is found in mostly large membrane complexes and localizes to dense vesicles, likely virus assembly zones late during infection. 

#### 2.3.2. HMCV US28 Is a Non-Glycosylated Membrane Protein

As integral membrane proteins, the extracellular portions of GPCRs have the potential to be glycosylated. Both US27 and UL33 are heavily glycosylated while present in infected cell membranes [10,11]. The US28 protein annotated from the HCMV genome contains a possible N-linked glycosylation site (amino acids 30–32, NQS). Therefore, to determine if US28 is a glycosylated membrane protein, lysates prepared from TB40/E-US28^YFP^-infected fibroblasts at various times post‑infection were subjected to digestion by either endoglycosidase H (EndoH) or peptide: N‑glycosidase F (PNGaseF) (Figure 3c). EndoH cleaves high mannose glycans from the core of N‑linked glycoproteins while PNGaseF hydrolyzes nearly all types of N-linked glycans from proteins [25]. Unexpectedly, US28^YFP^ was insensitive to cleavage by either EndoH or PNGaseF throughout the time course (Figure 3c, lanes 1–9), suggesting that US28 is not glycosylated despite being a membrane protein and trafficking through the secretory compartment [22]. As a control, the sensitivity of the known glycoprotein major histocompatibility complex (MHC) class I heavy chain to cleavage by EndoH and PNGaseF was determined (Figure 3c, lanes 10–18). Class I heavy chains traffic rapidly through the ER, where high-mannose glycans are acquired and cleaved, and is therefore resistant to EndoH digestion [26] (Figure 3c, lanes 11, 14, 17). Class I heavy chains were completely sensitive to cleavage by PNGaseF, resulting in loss of its single glycan (Figure 3c, lanes 12, 15, 18). The findings reveal that HCMV US28 is a unique non-glycosylated membrane protein. 

### 2.4. Functional Analysis of TB40/E US28 Variants

#### 2.4.1. TB40/E ΔUS28 Accumulates Increased Amounts of Extracellular Virus in Fibroblasts

**Figure 4:**
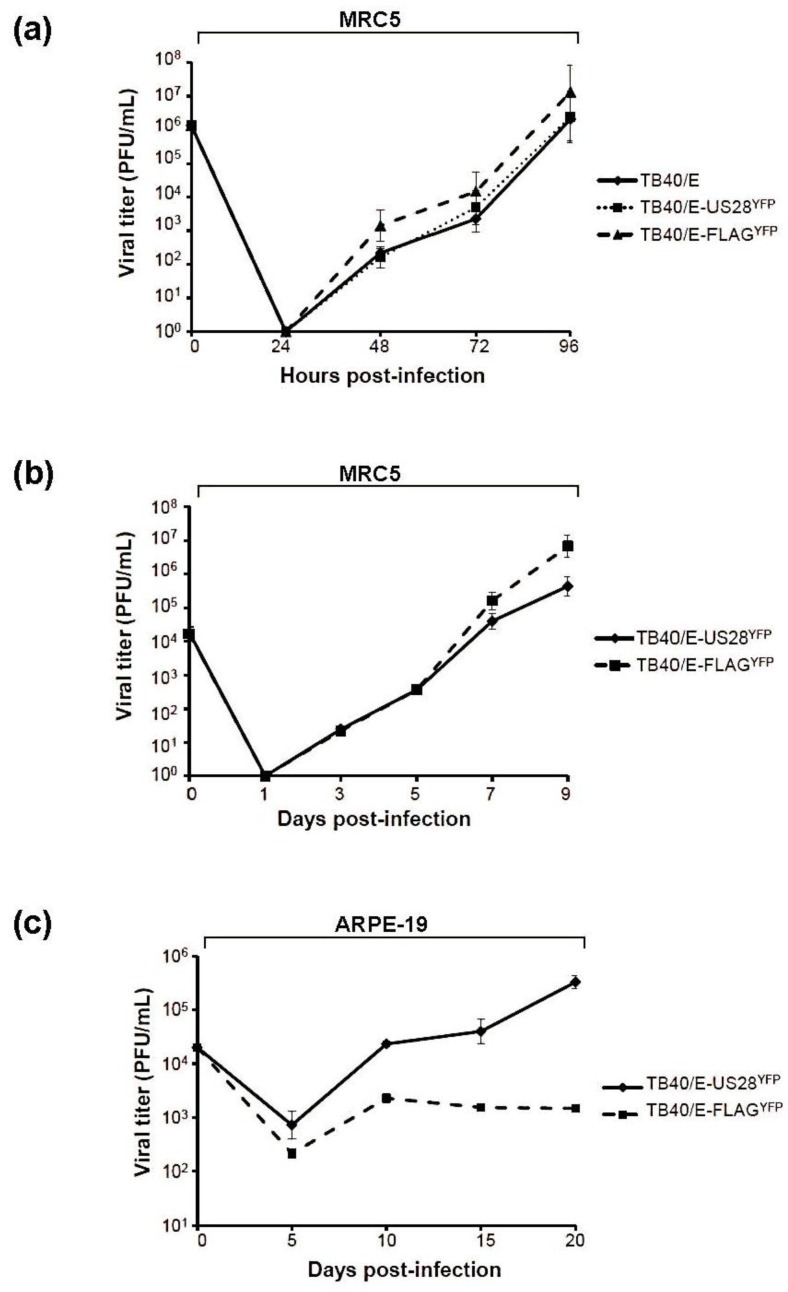
HCMV US28 modulates cell-to-cell spread of virus. Infectious extracellular progeny from fibroblasts infected at 5 PFU/cell (**a**) or 0.1 PFU/cell (**b**) with TB40/E wt, TB40/E-US28^YFP^, or TB40/E-FLAG^YFP^ were measured by TCID_50_ assay. Viral titers were assayed in duplicate. Error bars represent standard deviation of the mean. (**c**) ARPE-19 cells were infected at 0.1 PFU/cell with TB40/E-US28^YFP^ or TB40/E-FLAG^YFP^ and at the indicated times post-infection cell-associated virus was determined by TCID_50_ assay. Viral titers were assayed in duplicate. Error bars represent standard deviation of the mean.

To determine the growth properties of the TB40/E US28 variants, fibroblasts were infected at both high multiplicity, to study single step growth, and low multiplicity, to determine multi-step growth, and production of infectious extracellular progeny was measured (Figure 4a,b). At high multiplicity of infection (MOI), TB40/E wt and TB40/E-US28^YFP^ grew to comparable titers (Figure 4a, solid line *vs.* dotted line). In comparison, TB40/E-FLAG^YFP^ (ΔUS28) displayed a 10-fold increase in the accumulation of extracellular virus (Figure 4a, dashed line). This effect was amplified at low multiplicity of infection, where TB40/E-FLAG^YFP^ displayed a 100-fold increase in viral titers over TB40/E-US28^YFP^ (Figure 4b, solid line *vs.* dashed line). The data demonstrates that the loss of US28 results in increased production of extracellular virus during HCMV infection.

#### 2.4.2. US28 Modulates HCMV Cell-to-Cell Dissemination

An HCMV mutant virus lacking the tegument phosphoprotein pp28 fails to accumulate extracellular progeny yet mediates cell-to-cell spread of tegument-coated capsids [27]. The accumulation of extracellular virus seen during ΔUS28 infection could result from a blockade at the level of cell-to-cell spread, thus leading to re-routing of infectious virions into the extracellular milieu. To determine if the ΔUS28 virus has a defect in cell-to-cell spread of virus, infections were performed in epithelial cells, where spread of TB40/E is exclusively cell-associated [19]. ARPE-19 cells were infected at low multiplicity with either TB40/E-US28^YFP^ or TB40/E-FLAG^YFP^ and infectious progeny measured by determining the titer of cell-associated virus (Figure 4c). In comparison to fibroblasts, where TB40/E-FLAG^YFP^ displayed increased growth properties, the ΔUS28 virus exhibited a growth defect in epithelial cells (Figure 4c, dashed line). In fact, while titers of cell-associated TB40/E‑US28^YFP^ increased over the time course, accumulation of intracellular virus became stagnant during TB40/E-FLAG^YFP^ infection (Figure 4c, solid line *vs.* dashed line). Taken together, it appears that US28 may contribute to cell-to-cell dissemination during HCMV infection. 

### 2.5. Inhibition of TB40/E ΔUS28 Dissemination by Anti-HCMV Neutralizing Antibody

**Figure 5:**
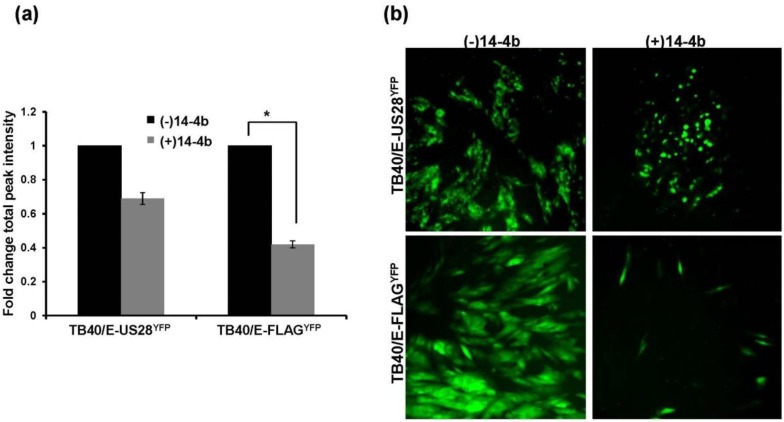
TB40/E ΔUS28 displays a growth defect when required to use the cell-to-cell route of dissemination. Fibroblasts infected (MOI = 0.01) with either TB40/E-US28YFP or TB40/E-FLAGYFP were cultured in the presence of the HCMV neutralizing antibody 14-4b. Two weeks post-infection cells were analyzed by fluorescent microplate reader (**a**) or by fluorescence microscopy (**b**). For (**a**) YFP fluorescence was assayed in triplicate. Error bars represent standard deviation of the mean; * *p* < 0.05, Student’s one tailed T-test.

When grown in fibroblasts TB40/E-FLAG^YFP^ produced substantial extracellular virus, suggesting that, in the absence of US28, the virus uses the extracellular route as the main route of dissemination. Therefore, an inhibition of infectious virus in the supernatant of ΔUS28-infected cells would result in a significant hindrance to dissemination. To determine if TB40/E-FLAG^YFP^ spread could be ablated in fibroblasts, infected cells were cultured in the presence of the HCMV neutralizing antibody 14-4b that recognizes glycoprotein H (gH) [28]. At approximately two weeks post-infection cells were analyzed by a fluorescence microplate cytometer (Figure 5a) and by fluorescence microscopy (Figure 5b). When grown in the presence of 14-4b, TB40/E-US28^YFP^ total fluorescence was slightly decreased (Figure 5a, left). In comparison, TB40/E-FLAG^YFP^ fluorescence was significantly reduced in the presence of 14-4b (Figure 5a, right). Fluorescence microscopy revealed that although TB40/E-US28^YFP^ grown in the presence of 14-4b created smaller plaques, cellular syncytia continued to be formed (Figure 5b). In contrast, TB40/E-FLAG^YFP^ growth was restricted to small pockets of infection in the presence of 14-4b, with the absence of plaque formation (Figure 5b). These findings demonstrate that US28 impacts cell-to-cell spread of virus as TB40/E-ΔUS28 is hindered in its dissemination in culture upon addition of HCMV neutralizing antibodies.

## 3. Experimental Section

### 3.1. Cells and Viruses

Human lung fibroblasts (MRC5) were maintained in Dulbecco’s modified Eagle’s medium (DMEM) containing 8% fetal bovine serum (FBS), 1 mM HEPES, 100 U/mL penicillin, and 100 μg/mL streptomycin. Human retinal pigmented epithelial cells (ARPE-19) were maintained in a 1:1 mixture of complete DMEM (FBS, HEPES, penicillin, streptomycin) and Ham’s F-12 medium containing 8% FBS, 1mM HEPES, 100 U/mL penicillin, and 100µg/mL streptomycin. Human umbilical vein endothelial cells (HUVEC) were cultured in EBM medium supplemented with bovine brain extract (BBE), recombinant human epidermal growth factor (rhEGF), hydrocortisone, ascorbic acid, gentamicin sulfate/amphotericin-B (GA-1000) and 2% FBS (Lonza Clonetics). CD34^+^ CD31^+^ human microvascular endothelial cells (HMVEC) were cultured in EBM-2 basal medium supplemented with rhEGF, hydrocortisone, recombinant human fibroblast growth factor-beta (rhFGF‑B), vascular endothelial growth factor (VEGF), insulin-like growth factor (R^3^-IGF-1), ascorbic acid, GA-1000, and 5% FBS (Lonza Clonetics). Cells were maintained at 37 °C in a humidified atmosphere (95% air/5% CO_2_). 

The HCMV bacterial artificial chromosome (BAC) clone of the clinical isolate TB40/E (TB40-BAC4) was a kind gift of Dr. Christian Sinzger (Institute of Virology, University Medical Center Ulm, Ulm, Germany) and Dr. Felicia Goodrum (University of Arizona, Tucson, Arizona, AZ, USA). TB40-BAC4 was used to generate variants expressing a chimeric US28 with a yellow fluorescent protein (YFP) tag (TB40/E-US28^YFP^) and a US28 deletion virus containing a FLAG-YFP cassette within the US28 open reading frame (TB40/E-FLAG^YFP^ (ΔUS28)). To generate these viruses TB40-BAC4 was modified using the galK recombineering system as previously described [29]. In brief, the galK gene was amplified by PCR using the following primers: Forward 5'-*GTGCGTGGACCAGGCGGTGTCCATGCACCGAGGGCAGAACTGGTGCTACCCCTGTTGACAATTAATCATCGGCA*-3'; Reverse 5'-*GAGGGGCGGACACGGGGTTTGTATGAAAAGGCCGAGGTAGCGATTTTTTATCAGCACTGTCCTGCTCCTT*-3', where the underlined sequences correspond to galK. The PCR product was transformed into recombination-competent E. coli SW105 cells containing TB40-BAC4. GalK-expressing clones were subsequently selected and electroporated with a PCR cassette specific to either US28^YFP^ or FLAG^YFP^ amplified from pcDEF-US28^YFP^. Primers used for reversion were as follows: US28^YFP^ Forward 5'-*GTGCGTGGACCAGGCGGTGTCCATGCACCGAGGGCAGAACTGGTGCTACCATGACACCGACGACGACGACCG*-3'; US28^YFP^/FLAG^YFP^ Reverse 5'-*GAGGGCGGACACGGGGTTTGTATGAAAAGGCCGAGGTAGCGCTTTTTTATTACTTGTACAGCTCGTCCATGC*-3'; FLAG^YFP^ Forward 5'-*GTGCGTGGACCAGGCGGTGTCCATGCACCGAGGGCAGAACTGGTGCTACCATGGACTACAAGGACGACGACGACACTAGTGCGGCCGCCATGGTGAGC*-3'. The resultant clones were chosen following counter-selection against galK and subsequently sequenced to ensure incorporation of YFP and FLAG. Virus stocks were generated by electroporating low passage MRC5s with purified BAC DNA from the respective variants. Cultures were allowed to progress to full cytopathic effect (CPE) and virus was then harvested and purified by centrifugation through a 20% sorbitol cushion. Virus stocks were stored at −80 °C in DMEM containing 8% FBS plus 1.5% bovine serum albumin (BSA). Virus stock titers were determined by tissue culture infectious dose 50 (TCID_50_) assay. 

### 3.2. Fluorescence Microscopy

For fluorescence microscopy, fibroblasts infected at a multiplicity of infection (MOI) of 5 plaque forming units (PFU)/mL were visualized 2 days post-infection using the EVOS Cell Imaging Systems (Life Technologies, Grand Island, NY, USA). Images were analyzed using Adobe Photoshop CS5.1 software [30]. ARPE-19 and endothelial cells were infected at an MOI of 25 PFU/mL and visualized 4 days post-infection. For confocal microscopy, fibroblasts infected at an MOI of 5 PFU/mL were harvested at various times post-infection and fixed in Cytofix/Cytoperm solution (BD Biosciences, Franklin Lakes, NJ, USA) for 45 minutes at 4 °C. YFP fluorescence was visualized using a Molecular Devices ImageXpress Ultra (IXU) plate-scanning confocal microscope (Integrated Screening Core, Icahn School of Medicine at Mount Sinai, New York, NY, USA). Images were analyzed using MetaExpress software [31]. 

### 3.3. Cell Fractionation and Immunoblot Analysis

Subcellular fractionation was performed as previously described [32]. In brief, mock-infected and TB40/E-infected fibroblasts were resuspended in 1× homogenization buffer (100 mm Tris, 150 mm NaCl, 250 mm sucrose, 1.5 mg/mL aprotinin, and 1 μM leupeptin) and mechanically homogenized using a 12-μm ball bearing homogenizer (Isobiotec, Hiedelberg, Germany). Samples were centrifuged at 15,000 × *g* for 10 minutes at 4 °C and heavy organelles found in the pellet were lysed directly in 1× SDS sample buffer (50 mm Tris, pH 6.8, 2% SDS, 10% glycerol, 0.02% bromphenol blue, 50 mm dithiothreitol). Supernatants were further centrifuged at 120,000 × *g* for 1 hour at 4 °C. Pellets containing cellular membranes and supernatants containing cytoplasm were lysed in SDS sample buffer and resolved using SDS-PAGE. Green fluorescent protein (GFP) polyclonal antibody was a kind gift of Dr. Hidde Ploegh (Whitehead Institute, MIT, Cambridge, MA, USA). Polyclonal major histocompatiblity class I heavy chain (MHC class I HC) antibody has been previously described [33]. Anti-glyceraldehyde-3-phosphate dehydrogenase (GAPDH) was purchased from Upstate/Millipore. Monoclonal glycoprotein B (gB) antibody was a kind gift of Dr. William Britt (UAB, Birmingham, AL, USA). 

### 3.4. N-Linked Protein Glycosylation Analysis

Endoglycosidase H (EndoH) and peptide: *N*-glycosidase F (PNGaseF) sensitivity was determined as per the manufacturer’s protocol (New England Biolabs, Ipswitch, MA, USA). In brief, polypeptide samples lysed in 1% SDS were incubated in 1× denaturing buffer (0.5% SDS, 0.04 m dithiothreitol) followed by the addition of 10× G5 buffer (0.5 m sodium citrate, pH 5.5, for EndoH) or 10× G7 buffer (0.5 m sodium citrate, pH 7.5, 10% Nonidet P-40, for PNGaseF) and 1000 units of EndoH or 500 units of PNGaseF. Enzymatic reactions were carried out at 37 °C for 2 hours. 

### 3.5. Analysis of Virus Growth and Spread

Single step growth kinetics were determined by infecting fibroblasts at an MOI of 5 PFU/mL. At the indicated time points post-infection media was collected and virus titers in the supernatant were determined by TCID_50_ assay. Multistep growth kinetic analysis was performed at an MOI of 0.1 PFU/mL. For analysis of cell-associated virus yield, ARPE-19 cells were infected at an MOI of 0.1 PFU/mL and samples harvested by scraping cells into media. Cells were lysed by subjecting them to a single freeze-thaw cycle and sonication. Cellular debris was pelleted by centrifugation and the amount of infectious virus in the resulting supernatant was determined by TCID_50_ assay.

For neutralization of extracellular virus, fibroblasts were mock-infected or TB40/E-infected at an MOI of 0.01 PFU/mL for 1 hour at 37 °C and then placed into media containing 10 µg/mL of the monoclonal anti-gH antibody 14-4b (a kind gift of Dr. William Britt (UAB)). 2 weeks post-infection YFP fluorescence was analyzed using an Acumen ^e^X3 laser scanning fluorescence microplate cytometer (TTP LabTech, Cambridge, MA, USA) as previously described [34]. 

## 4. Conclusions

Many functions have been ascribed to the G protein-coupled receptors encoded by HCMV. In these studies we demonstrate that US28 impacts dissemination of virus by promoting cell-to-cell spread of infectious progeny. The generation of two fluorescent variants of the clinical isolate TB40/E, US28^YFP^ and FLAG^YFP^ (ΔUS28), allowed us to investigate the role of US28 as a virulence factor during HCMV infection (Figure 1). Infection with TB40/E-US28^YFP^ resulted in the formation of intense perinuclear granular structures at late times in fibroblasts, endothelial, and epithelial cells (Figure 2). Interestingly, in epithelial cells, where TB40/E disseminates via the cell-to-cell route, US28^YFP^ localized to areas between infected cells (Figure 2b). During infection, US28^YFP^ traffics with mostly large dense complexes in the membranes of infected cells (Figure 3a,b). Surprisingly, US28^YFP^ did not acquire an N-linked glycan during infection (Figure 3c), ascribing a novel characteristic to this viral membrane protein. Infections with TB40/E-US28^YFP^ resulted in growth properties comparable to TB40/E wt (Figure 4a). Strikingly, infection with TB40/E-FLAG^YFP^, a virus lacking US28, resulted in increased production of extracellular infectious progeny (Figure 4a,b). However, when assayed in epithelial cells, where dissemination occurs via cell-to-cell spread, TB40/E-FLAG^YFP^ demonstrated a growth defect (Figure 4c) suggesting that US28 plays a role in inter-cellular dissemination of HCMV. Accordingly, when the virus lacking US28 was forced to utilize the cell-to-cell route in fibroblasts by culture in the presence of neutralizing antibodies, a substantial defect in dissemination was observed (Figure 5). Taken together, we can conclude that US28, an integral membrane protein present at the border of adjacent cells, plays a role in dissemination of infectious viral progeny from cell-to-cell. 

The scenario may occur during HCMV infection where, at late time points, US28 converges at the interface of viral assembly zones and adjoining cells in order to enhance virus spread to uninfected neighboring cells. The dissemination enhancing activity of US28 likely requires the interaction of this GPCR with a membrane component on adjacent cells. Due to US28’s high affinity binding to the chemokine fractalkine [35], this interaction might either promote cell-cell contact or membrane fusion in clinically relevant cell types expressing this CX_3_C membrane-bound chemokine (*i.e.*, endothelial cells). In fact, fusion-enhancing activity has been reported for US28 when expressed together with the HIV Env protein and the G protein of vesicular stomatitis virus (VSV-G) [36]. Heteromerization of US28 and other HCMV GPCRs [37] may alter the physical properties of membranes in a way favorable to fusion with neighboring infected cells. Interestingly, HCMV US27 and UL33 or MCMV M33 did not seem to enhance cell fusion in a way comparable to US28 [36], suggesting a novel role for this GPCR in dissemination of HCMV in the host. The pattern of US28^YFP^ trafficking and its membrane association would be well-suited to incorporation of this protein into the viral lipid envelope. As a component of the virion, US28 could have a direct role in HCMV entry through its ability to bind target cells and mediate dissemination. This would be most advantageous during latent infection of HCMV when US28, shown to be expressed during latency [38], could mediate adhesion of latently-infected monocytes to activated endothelial cells, where high levels of fractalkine are expressed [39], to potentiate dissemination in the host. Conceivably, virion-bound GPCR US28 could also bind to cells and induce signaling networks to facilitate infection. However, further experiments are necessary to define whether US28 is present in virions and whether it can play a role in virus-cell interaction. 

HCMV dissemination can occur via two routes: through production of extracellular virus progeny that subsequently bind and enter new cells to propagate infection or directly from cell to cell with limited exposure of virus to the extracellular environment [27]. Here we define US28 as a factor contributing to cell-to-cell dissemination of HCMV. Our data strongly supports the role of viral-encoded GPCRs as virulence factors *in vitro* and further adds to our understanding of the multifunctional US28 protein. We also identify US28 as a novel target for anti-HCMV therapeutics to inhibit viral dissemination in the host. 
